# Predictors of Participant Attendance Patterns in a Family-Based Intervention for Overweight and Obese Hispanic Adolescents

**DOI:** 10.3390/ijerph15071482

**Published:** 2018-07-13

**Authors:** Sara M. St. George, Mariya Petrova, Tae Kyoung Lee, Krystal M. Sardinas, Marissa A. Kobayashi, Sarah E. Messiah, Guillermo Prado

**Affiliations:** 1 Department of Public Health Sciences, University of Miami Miller School of Medicine, Miami, FL 33136, USA; mpp72@med.miami.edu (M.P.); txl371@med.miami.edu (T.K.L.); k.sardinas@med.miami.edu (K.M.S.); mxk748@med.miami.edu (M.A.K.); SMessiah@med.miami.edu (S.E.M.); gprado@med.miami.edu (G.P.); 2 Department of Pediatrics, University of Miami Miller School of Medicine, Miami, FL 33136, USA

**Keywords:** attendance, family, obesity, intervention, Hispanic, acculturation

## Abstract

This study examined participant attendance patterns and individual (e.g., income), family dynamics (e.g., communication), and cultural (i.e., Americanism, Hispanicism) predictors of these patterns among Hispanic families enrolled in a 12-week family-based intervention, Familias Unidas for Health and Wellness. Hispanic adolescents (*n* = 140, 49% female, 13.04 ± 0.87 years old, 36% overweight, 64% obese, 39% immigrants) and their parents (87% female, 42.09 ± 6.30 years old, BMI 30.99 ± 6.14 kg/m^2^, 90% immigrants) were randomized to the intervention condition. A repeated measures latent class analysis that included 12 binary variables (yes/no) of attendance identified three subgroups of attendance patterns: consistently high, moderate and decreasing, and consistently low. An ANOVA was then conducted to examine whether the identified attendance patterns differed by individual, family dynamics, and cultural characteristics at baseline. Parents in the consistently high attendance group had lower Americanism than those in either of the other attendance groups. Adolescents in the consistently high attendance group had lower Hispanicism than those in either of the other attendance groups. No other variables significantly discriminated between attendance groups. Sustained attendance in the Familias Unidas for Health and Wellness intervention may be driven by Hispanic parents’ desire to better understand their host culture, connect with other culturally similar parents, and reconnect adolescents with their heritage culture.

## 1. Introduction

Pediatric obesity is one of the most significant preventable public health problems in the United States [1]. It has been consistently associated with poor physical (e.g., type 2 diabetes, cardiovascular disease) and psychological outcomes (e.g., mood disorders) later in life [2,3,4]. Thirty-five percent of youth aged 2–19 years old are currently classified as overweight or obese (Body Mass Index (BMI) for age and gender ≥ 85th percentile), with data from the 2015–2016 cycle of the National Health and Nutrition Examination Survey (NHANES) highlighting persisting health disparities for Hispanic youth [5]. Specifically, Hispanics have the highest overall rates of pediatric overweight and obesity (46%) compared to non-Hispanic black (38%), white (30%), and Asian (23%) youth [5]. These rates correspond to low levels of physical activity and poor dietary quality [6,7,8]. Developing, evaluating, and disseminating evidence-based obesity prevention interventions for Hispanics, estimated to comprise 28% of the U.S. population by the year 2060 [9], is thus critical to the future health of the nation.

The family may be the most fundamental social system influencing child health and development [10,11], and is especially relevant for Hispanics due to *familismo* (i.e., a Hispanic cultural value placing a strong emphasis on family loyalty and commitment) [12]. A recent systematic review of obesity-related randomized controlled trials conducted with Hispanic youth aged 5–19 years old identified 11 interventions published between 2010–2015, the majority of which (72%) included some sort of family-based component [13]. Although family-based pediatric weight loss studies have been found to successfully reduce youth BMI [14], stronger effects are often detected in those with higher versus lower rates of attendance [15,16,17]. Notably, poor attendance in both clinical and research settings has been cited as one of the most common challenges of pediatric obesity interventions, with the highest rates of attrition observed among low income and ethnic minority populations [18,19,20,21,22]. Understanding participant attendance patterns, as well as factors predicting those patterns, is therefore necessary to implement strategies that will maximize the public health impact of family-based obesity prevention programs, particularly among Hispanic youth at greatest risk for being overweight and obese.

### 1.1. Intervention Attendance: Rates versus Patterns

Most studies investigating intervention attendance (and predictors thereof) in the field of pediatric obesity have operationalized it as a sum (total number of sessions attended) or a rate (percentage of total sessions attended), e.g., References [23,24]. While informative, such approaches are limited in that they do not provide important temporal information regarding participant attendance patterns and trajectories over the course of an intervention. As noted by Mauricio et al. [25,26], understanding attendance trajectories and profiling families based on these patterns may help interventionists identify when and for whom declines in attendance are likely to occur.

Examples of studies that capture attendance more dynamically as a pattern over time can be found in the broader family-based prevention science literature [25,26,27,28,29]. For instance, a study of attendance patterns among mothers and fathers enrolled in a 10-session parenting-focused preventive intervention for divorced/separated parents used a latent class growth analysis to identify four classes of attendance: non-attenders, early dropouts, declining attenders, and sustained attenders [25]. This study found that mothers who were early dropouts were more likely to be Hispanic and report higher parental conflict than those who were sustained attenders [25]. Similar classes of attendance have been observed in other family-based preventive interventions targeting youth internalizing, externalizing, and risky behaviors (e.g., substance use, sexual risk-taking) among exclusively Hispanic samples [26,28,29]. To our knowledge, however, no prior obesity-related intervention studies targeting Hispanic youth have examined participant attendance patterns.

### 1.2. Predictors of Participant Attendance

Understanding what predicts participant attendance in family-based interventions is complex, as attendance may be influenced by a broad range of variables, including individual characteristics of both the target youth and their parents, family dynamics, and the broader cultural variables, e.g., References [23,24,30,31]. Our approach to examining predictors of attendance was therefore grounded in the eco-developmental framework [32]. The eco-developmental framework is a contextual schema that organizes risk and protective factors across multiple ecological systems. It draws from social ecological theory [33], which emphasizes that human behavior is influenced by factors ranging from those most proximal to the individual (i.e., microsystems, or contexts in which the individual participates directly) through to those more distal to the individual (i.e., macrosystem, or the broader cultural and societal context). In this study, we examined predictors of attendance patterns in a family-based obesity prevention intervention across three levels: individual (adolescent and parent), family, and the broader cultural context.

#### 1.2.1. Individual-Level Predictors of Attendance

Strong evidence suggests sociodemographic factors impact attendance, with higher levels of parental education, greater household income, and partnered (vs single) parenting consistently associated with higher attendance and retention in family-based obesity interventions [18,23,24,34,35,36]. Families with girls versus boys have also been shown to be more likely to initiate and complete obesity-related programs [35,36]. Other individual-level variables, such as the health status and lifestyle behaviors of both parents and youth at baseline may also be associated with attendance, though relatively little research has examined these associations. In a study that investigated demographic and psychosocial predictors of attrition in a family-based weight control trial among adolescents aged 13–16 years old, for example, parent BMI was the most robust predictor of attrition [37]. Specifically, families with a parent who had a higher versus lower BMI were 4.6 times more likely to drop out of the intervention. Another study found that the child’s healthy eating at baseline was positively associated with attendance in a family-based lifestyle intervention for overweight children [24]. Together, these studies suggest that families of adolescent girls who have more resources/support and are physically “healthier” at intervention onset may be more likely to have sustained attendance.

#### 1.2.2. Family-Level Predictors of Attendance

Other key factors potentially influencing attendance in family-based pediatric obesity interventions may be related to the functioning of the family unit. For example, Williams and colleagues [18] found that those classified as program non-completers and partial completers had higher levels of family dysfunction (e.g., family members avoiding each other when at home) than did those classified as program completers. Due to the limited number of studies examining the influence of family variables on attendance in obesity-related studies, we drew from the broader family-based prevention science literature. Family-level variables, including poorer family communication, lower family organization (i.e., the degree of order in the home), and higher levels of conflict, have been previously associated with an increased likelihood of intervention dropout [29], poorer initial engagement (defined as attending one of the first three sessions) [38], and lower program participation and retention [31,39,40]. These or similar family variables, including low use of effective parenting skills, however, have also been found to positively predict attendance in family-based substance use and problem behavior preventive interventions due to potentially being associated with a greater intervention need [30,41,42,43]. Overall, more research that examines the effects of family-level variables on attendance in obesity prevention interventions is needed.

#### 1.2.3. Cultural Level Predictors of Attendance

The family-based prevention literature additionally points to acculturation as an important cultural factor to consider in interventions targeting Hispanic immigrant families. Acculturation, or the processes of immigrants adapting to their host country’s culture, may influence a family’s willingness to participate in an intervention program [44]. Less acculturated individuals, or those who identify more strongly with their culture of origin and less strongly with their host culture, tend to participate in culturally syntonic family-based interventions at higher rates [26,28,30,44]. In a recent study of attendance patterns among Mexican-Americans participating in a mental health and substance use prevention program, for example, those with lower versus higher levels of acculturation were more likely to belong to the sustained attendance group [28]. Although studies with more diverse Hispanic samples have found similar associations [30], these studies are not as well-represented in the literature as are those with exclusively Mexican-American samples.

### 1.3. Current Study and Hypotheses

In the context of a culturally relevant family-based obesity prevention intervention for Hispanic adolescents known as Familias Unidas for Health and Wellness [45], the aims of the current study were to: (1) identify subgroups of participant attendance patterns, and (2) examine the role of individual (i.e., adolescent gender, parent education, annual household income, adolescent and parent weight status and healthy lifestyle behaviors), family dynamics (i.e., positive parenting, parent-adolescent communication), and cultural variables (i.e., acculturation, Hispanicism, Americanism) in distinguishing attendance patterns. We hypothesized that multiple attendance patterns would emerge from our data and that families with the following characteristics at baseline would be most likely to consistently attend intervention sessions: female adolescent gender, higher parent education, higher annual household income, lower parent and adolescent weight status, healthier adolescent and parent lifestyle behaviors, more positive parenting, better parent-adolescent communication, and less acculturation.

## 2. Materials and Methods

### 2.1. Participants

Participants were made up of 140 adolescents (49% female, 13.04 ± 0.87 years old, 36% overweight, 64% obese, 39% immigrants) and their parents (87% female, 42.09 ± 6.30 years old, 52% obese, 58% married, 69% < $30 K annual household income, 90% immigrants) randomly assigned to the Familias Unidas for Health and Wellness intervention condition. A total of 140 other participants were randomized to a comparison condition, but they were not included in this analysis. See Table 1 for participant demographic characteristics. Families were eligible to participate if they had a Hispanic adolescent who (1) was in the 7th or 8th grade, (2) had a BMI ≥ 85th percentile for their age and sex [46], and (3) lived with an adult primary caregiver willing to participate in the two-year study. Families were excluded if they planned to move out of South Florida during the two-year study period. In addition, if parent responses on a physical activity readiness questionnaire indicated a serious health issue (e.g., chest pain) for parents and/or adolescents, they were required to acquire physician approval to participate.

Study staff recruited participants from 18 middle schools in Miami-Dade County. Schools were selected based on their proximity to county parks where the intervention would be held and were concentrated in two areas in South Florida: a middle class, more established area and a lower-income area with a higher concentration of recently-arrived immigrants. Study staff first distributed letters describing the study to 7th and 8th grade classrooms. Letters instructed interested parents to provide their contact information and informed them that study staff would potentially call with additional details. As students returned signed letters, study staff visually screened adolescents to identify those who might have a BMI ≥ 85th percentile for their age and sex based on body silhouette images [47]. This procedure was used to reduce the stigma associated with obesity (i.e., letters did not mention the BMI criterion and described the intervention broadly as one designed to “promote healthy choices and prevent risky behaviors in Hispanic youth”). A doctoral level exercise physiologist trained all study staff to identify potentially eligible youth using the body silhouette images. Within one week of collecting signed letters, study staff contacted only parents of potentially eligible adolescents by phone and invited them to participate. During these phone calls, study staff highlighted the upcoming adolescents’ transition to high school and emphasized that the intervention would promote healthy adolescent choices across a range of outcomes (e.g., physical health, substance use, sexual risk-taking). Final eligibility for the BMI criterion was determined using objective measures of height and weight taken as the baseline assessment, usually scheduled within one week of the phone call.

### 2.2. Procedure

The Institutional Review Boards at the University of Miami and the Miami-Dade County Public School system approved this study. Trained study staff blind to condition assignment collected parental informed consent and adolescent assent and subsequently took objective measures of adolescent height and weight. If the adolescents’ BMI was <85th percentile for age and gender, the family was not eligible to participate. If the adolescent’s BMI was ≥85th percentile for age and gender, the family was eligible to participate and therefore completed the remaining baseline measures. After data collection, participants were randomized to Familias Unidas for Health and Wellness (experimental condition) or a community practice (comparison condition) using urn randomization [48] and concealment of allocation procedures (i.e., participants chose a sealed, opaque envelope from a box at random determining their condition assignment). Parents received $50 for completing the baseline data collection, and adolescents received one movie ticket.

### 2.3. Intervention Description

Familias Unidas for Health and Wellness was adapted from an evidence-based intervention for Hispanic adolescents (“Familias Unidas”) shown to be efficacious and effective in preventing and reducing substance use and sexual risk behaviors in Hispanic adolescents across four randomized controlled trials [49,50,51]. The adaptation process is described in detail elsewhere [45]. Briefly, intervention developers and experts in pediatric obesity, exercise physiology, dietetics, and the local parks system provided input for changes. The study team collected qualitative data from Hispanic adolescent–parent dyads before, during, and after an initial pilot test of the adapted intervention, and made adaptations to complement and enhance the original intervention. The adapted intervention maintained fidelity to Familias Unidas’ core theoretical elements and overall structure, but included additional content focused on physical activity and nutrition, adolescent participation in physical activity, and joint parent–adolescent participation in physical activity and nutrition skill-building activities.

Familias Unidas for Health and Wellness was a selective and indicated preventive intervention that aimed to improve physical activity levels and quality dietary intake in overweight and obese adolescents. The intervention was comprised of 12 sessions (eight 2.5 h group and four 1 h family sessions) delivered in the following sequence: family session 1, group sessions 1–2, family session 2, group sessions 3–5, family session 3, group sessions 6–7, family session 4, group session 8. See Table 2 for weekly intervention session topics and activities. During the first 1.5 h of group sessions, parents and adolescents participated in separate activities. Parents engaged in facilitator-led discussions and role-plays related to adolescent healthy lifestyle behaviors (i.e., physical activity, nutrition), risky behaviors (e.g., drugs/alcohol, risky sexual activity), and positive parenting strategies (e.g., family communication, parental involvement and monitoring, behavior management). Two bilingual facilitators trained in using problem-posing and participatory learning (e.g., eliciting feedback, placing parents in charge of the intervention’s direction) led these sessions. Meanwhile, adolescents engaged in outdoor physical activities led by local park coaches trained in the Sports, Play, and Active Recreation physical activity program, an evidence-based physical activity afterschool program (SPARK) [52]. During the second hour of group sessions, parents and adolescents engaged in a joint group activity meant to promote either physical activity or healthy nutrition. These interactive activities included hands-on nutrition education and training (i.e., cooking) led by a local non-profit organization, a park obstacle course, and fitness classes (i.e., yoga, Zumba) taught by certified instructors. The purpose of the group activities was to provide parents with an opportunity to practice the skills learned during the first half of the group sessions with their adolescents and to demonstrate entertaining, healthy activities parents and adolescents can do together.

During the four family sessions, facilitators met individually with each family and encouraged parents to practice the skills they learned within the group sessions. Parents and adolescents engaged in guided discussions and role-plays related to adolescent lifestyle behaviors and risky behaviors.

### 2.4. Measures

#### 2.4.1. Participant Attendance

Intervention facilitators recorded participant attendance at each of the eight group sessions and four family sessions. Attendance was coded as either 0 = family not in attendance or 1 = family in attendance. Families attended an average of 71% of all 12 sessions. Overall, 5% of families did not attend any intervention sessions, 11% attended 1–3 sessions, 11% attended 4–6 sessions, 17% attended 7–9 sessions, 26% attended 10–11 sessions, and 30% attended all 12 sessions

#### 2.4.2. Anthropometric Measures

Study staff measured participants’ height and weight at baseline using a SECA 217 mobile stadiometer and a SECA 869 digital scale (SECA, Hamburg, Germany), respectively. To improve the accuracy of these measures, study staff instructed participants to remove their shoes, empty their pockets, and remove any other bulky clothing and accessories. We calculated adolescent BMI percentiles using the most recently available Centers for Disease Control and Prevention growth reference curves [46] and adult BMI using the following standardized fraction: BMI = weight (kg)/height (m^2^).

#### 2.4.3. Participant Self-Reported Measures

Parents and adolescents completed a computer-based questionnaire using the Audio Computer-Assisted Self-Interview software, an electronic data collection system [53]. The survey was available in English or Spanish, and participants had the option to listen to the questions using headphones.

##### Demographics

Parents reported their level of education by identifying the highest grade they completed in school; response options ranged from first grade through to the completion of a graduate or professional degree. They additionally reported their current annual household income, with response options in $5000 intervals ranging from <$5000 to ≥$50,000.

##### Physical Activity

Parents and adolescents responded to a single item on the NHANES Physical Activity and Physical Fitness Questionnaire [54]: “During the past 7 days, on how many days were you physically active for a total of at least 60 min per day? Add up all the time you spent in any kind of physical activity that increased your heart rate and made you breathe hard some of the time”. Response options ranged from 0–7 days.

##### Fruit, Vegetable, and Added Sugar Intake

Parents and adolescents completed the NHANES Dietary Screener Questionnaire [55]. This 26-item screener asked participants how often in the past month they consumed specific foods and drinks (e.g., fruits and vegetables, added sugars, whole grains/fiber, meat). Response options range from “Never” to “2 or more times per day”. Example questions included: “During the past month, how often did you eat fruit? Include fresh, frozen, or canned fruit. Do not include juices”, and “During the past month, how often did you eat ice-cream or other frozen desserts? Do not include sugar-free kinds”. A fruit and vegetable variable was calculated based on participants’ responses to the following specific food items: fruit, fruit juice, salad, fried potatoes, other potatoes, dried beans, other vegetables, tomato sauce, salsa, and pizza. An added sugars variable was calculated based on participants’ responses to the following specific food items: soda, fruit drinks, cookies, cake, pie, doughnuts, ice cream, sugar/honey in coffee/tea, candy, cereal, and cereal type. We used scoring algorithms developed by the National Cancer Institute to calculate cup equivalents per day of fruits and vegetables and teaspoon equivalents per day of added sugars [56].

##### Positive Parenting

Parents and adolescents completed the positive parenting subscale of the Parenting Practices Scale [57]. This nine-item subscale assessed how often over the past six months parents acknowledged and rewarded their adolescents in response to positive behaviors using a 5-point Likert scale ranging from 0 = “Never” to 4 = “Always”. A sample parent item is: “When your child has done something that you like or approve of, do you say something nice about it, praise or give approval?” A sample adolescent item is: “When you have done something that your parents like or approve of, how often does your mother/father do something special together with you, such as going to the movies, to a game, playing a game or going somewhere special?” We calculated a single score of positive parenting separately for parents and adolescents by summing across the nine items. Cronbach’s alpha was 0.60 for parents and 0.68 for adolescents.

##### Parent–Adolescent Communication

Parents and adolescents completed the Parent–Adolescent Communication Scale [58]. This 20-item measure assessed the quality and content of communication between parents and adolescents using a 5-point Likert scale ranging from “1 = Strongly Disagree” to “5 = Strongly Agree”. A sample parent item is: “I find it easy to discuss problems with my child”. A sample adolescent item is: “It is very easy for me to express all my true feelings to my mother”. We calculated a single score of parent–adolescent communication separately for parents and adolescents by summing across the 20 items. Cronbach’s alpha was 0.84 for parents and 0.84 for adolescents.

##### Acculturation (Hispanicism, Americanism)

Parents and adolescents completed the Bicultural Involvement Questionnaire, a scale designed specifically for Hispanics that assessed the degree to which one feels comfortable in two cultures: Hispanic and Anglo-American [59]. The scale included two 11-item subscales that assessed Hispanicism and Americanism, respectively, using a 5-point Likert scale ranging from “1 = Not at all Comfortable” to 5 = “Very Comfortable”. Sample items from the Hispanicism subscale included: “How comfortable do you feel speaking Spanish in general?” and “How much do you enjoy Hispanic music?” Sample items from the Americanism subscale included: “How comfortable do you feel speaking English with friends?” and “How much do you enjoy American TV programs?” We calculated scores for Hispanicism and Americanism separately for parents and adolescents by summing across the two 11-item subscales. Chronbach’s alphas for the Hispanicism and Americanism subscales were 0.82 and 0.94 for parents and 0.86 and 0.87 for adolescents, respectively.

### 2.5. Data Analysis

The aims of this study were to: (1) identify unobserved subgroups of participant attendance patterns in the Familias Unidas for Health and Wellness intervention, and (2) explore associations between eco-developmental risk and protective factors and subgroups of attendance patterns. As such, the analytic plan included two steps. First, we used a repeated measures latent class analysis (RMCLA) [60] that included 12 binary variables (yes/no) of participant attendance across each of the 12 intervention sessions to identify subgroups of attendance patterns. RMCLA is a special type of Growth Mixture Model (GMM) [60]. GMMs use a latent mixture approach to model a repeated outcome’s development (intercept and slope) and typically require equally-distributed time intervals (e.g., four or five time points with equal time intervals between points). When time intervals are not identical across participants (as they are in the present study), growth factors (i.e., intercept and slope) cannot be estimated. As such, we used RMCLA to estimate free slope factors defined by the repeated variables, rather than by estimated growth factors [61]. This approach was more appropriate to estimate classes of attendance patterns in this study.

We used the following fit statistics to inform the number of classes: (1) information criterion statistics (Akaike Information Criterion (AIC), Bayesian Information Criterion (BIC), Sample-size adjusted BIC (SSABIC)), where lower values indicate better fit; (2) the Adjusted Lo-Mendell-Rubin likelihood ratio test (Adj. LMR-LRT) [62], which provides a *p*-value indicating whether the estimated model provides a significantly better fit to the data than a model with one less class; (3) average posterior probability, which when ≥0.70 implies satisfactory fit for clear class assignment [63]; (4) entropy values, with values close to 1 indicating greater clarity in classification [64]; (5) sample size in each class, with a minimum of 5% for the smallest class [65]; and (6) overall interpretability of identified classes. To find the optimal class solution, we estimated models with one to five classes using a full-information maximum likelihood (FIML) estimator with robust standard errors in Mplus (Version 7.2, Muthén & Muthén, Los Angeles, CA, USA) [66].

In the second step of the analysis, we conducted an analysis of variance (ANOVA) to examine whether identified attendance patterns in the Familias Unidas for Health and Wellness intervention differed by participants’ eco-developmental risk and protective factors at baseline. To conduct an ANOVA examining the profiles of class membership, we exported class memberships to SPSS (Version 24.0, IBM, Armonk, NY, USA) once we had selected the optimal class model and used a least squares difference tests approach to run these post-hoc analyses.

## 3. Results

### 3.1. Identification and Interpretation of Participant Attendance Patterns

As can be seen in Table 3, a RMCLA using twelve binary variables (yes/no) of participant attendance across each of the 12 intervention sessions suggested that the 3-class solution provided a better fit to the data than did the 2-, 4-, or 5-class solutions. Although 4- and 5-class solutions showed slightly smaller IC values and acceptable entropy values compared to the 3-class solution, the *p*-value of the Adj. LMR-LRT was not significant, indicating that the 3-class solution was an adequate fit. Furthermore, the class size for the 3-class solution was acceptable.

**Figure 1:**
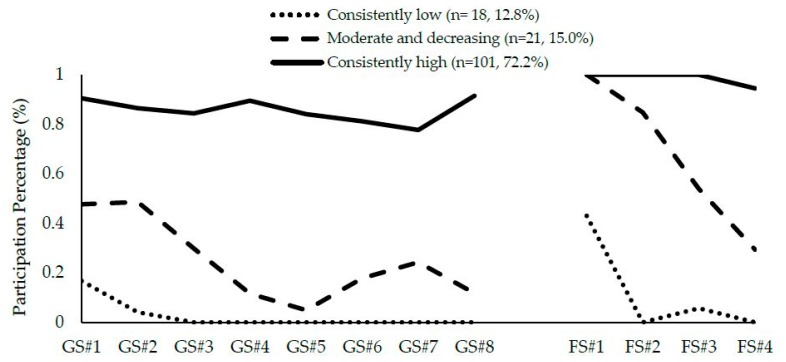
Heterogeneity in attendance patterns for Familias Unidas for Health and Wellness. GS = group session, FS = family session.

We examined the plot to see whether the results of the 3-class solution made interpretive sense. As can be seen in Figure 1, all three attendance patterns were clearly distinct. For example, the first class (*n* = 18, 13%) showed that attendance was stable and low across all group and family sessions (labeled as “consistently low” attendance). Participants in this class largely withdrew from the intervention, attending an average of only 0.72 ± 0.67 sessions. The second class (*n* = 21, 15%) indicated moderate attendance at the first group sessions that then decreased. This group also showed a similar decrease in attendance for the family sessions (labeled as “moderate and decreasing” attendance). Participants in this class attended an average of 4.5 ± 1.25 sessions. The last class (*n* = 101, 72%) was characterized by high attendance across all group and family sessions (labeled as “consistently high” attendance). Participants in this class attended an average of 10.7 ± 1.37 sessions.

### 3.2. Predictors of Participant Attendance Patterns

Results of the ANOVA indicated that there were significant mean differences among two variables predicting attendance class membership: parents’ Americanism and adolescents’ Hispanicism (see Table 4). The means of parents’ Americanism was significantly different among the three attendance patterns (F = 3.91, *p* < 0.05). Parents in the consistently high attendance group had lower reported levels of Americanism (35.54 ± 11.78) compared to those in the consistently low attendance group (37.08 ± 12.70) and in the moderate and decreasing attendance group (43.42 ± 10.73). We detected a similar significant mean difference regarding adolescents’ Hispanicism (F = 3.07, *p* < 0.05). Adolescents in the consistently high attendance group had lower reported levels of Hispanicism (36.84 ± 8.56) compared to those in the consistently low attendance group (41.50 ± 9.26) and in the moderate and decreasing attendance group (40.19 ± 8.24). There were no other significant mean differences among variables in our model predicting attendance class membership.

## 4. Discussion

This study examined participant attendance patterns as well as individual, family dynamics, and cultural predictors of these patterns among 140 Hispanic families enrolled in a 12-session family-based obesity prevention intervention. Results indicated the presence of three distinct attendance patterns: consistently low (average of <1 session attended), moderate and decreasing (average of 4.5 of 12 sessions attended), and consistently high (average of 11 of 12 sessions attended), with the overwhelming majority of participants in the consistently high attendance group. Parents in the consistently high attendance group reported lower Americanism than parents in either the moderate and decreasing or consistently low attendance groups. For adolescents, those in the consistently high attendance group reported lower Hispanicism than those in either the moderate and decreasing or consistently low attendance groups. No other baseline variables, including adolescent gender, parent education, annual household income, adolescent and parent weight status, adolescent and parent healthy lifestyle behaviors (i.e., physical activity, fruit and vegetable intake, added sugar intake), positive parenting, and parent-adolescent communication, significantly discriminated between attendance groups. Results suggested that sustained attendance in the Familias Unidas for Health and Wellness intervention may be driven by Hispanic parents’ desire to better understand their host culture, connect with other culturally similar parents, and reconnect their adolescents with their heritage culture.

To our knowledge, this is the first study in the field of obesity prevention to examine attendance as a pattern, rather than as a sum or a rate, e.g., References [23,24]. Visually examining the attendance patterns presented in Figure 1 reinforced the importance of attendance at the first few sessions, particularly among those in the moderate and decreasing attendance group, whose attendance to group sessions was modest from the outset but then steeply declined following the second group session. This type of information is inherently missing from studies that present attendance as a sum or a rate and may be used to determine optimal times for encouraging or incentivizing participants to continue attending sessions.

Notably, the majority of participants randomized to the intervention condition (72%) belonged to the consistently high attendance group, suggesting that once most families began the intervention, they continued returning to sessions. A recent review of barriers and facilitators to initial and continued attendance in community-based lifestyle programs for overweight and obese youth concluded that a family-centered approach (where both parents and youth attend sessions), practical sessions (e.g., hands on cooking activities and physical activity), and social interaction and support were important factors influencing continued attendance [67]. All of these characteristics are central to the present intervention and were previously endorsed by those participating in the initial pilot/feasibility study as important features of the program [45]. Specifically, parents described feeling engaged by intervention content and social connections, reported enjoying hands-on nutrition training activities, perceived improvements in family cohesion, and described the intervention social climate as being very positive.

Beyond identifying patterns of attendance, we examined a series of eco-developmental variables at baseline that might distinguish between the attendance groups. Unlike previous obesity-related studies [24,35,36], sociodemographic variables, namely parent education, annual household income, and marital status did not significantly predict participants’ attendance group. This finding suggested that in a culturally-specific intervention for Hispanics like Familias Unidas for Health and Wellness, socioeconomic status (SES) may not be as important for attendance as it is in interventions that are not culturally syntonic or that do not target specific ethnic groups. Although not significant, it is noteworthy that those in the consistently low attendance group reported the lowest income and highest levels of Hispanicism. Because we recruited participants from two somewhat distinct areas (a middle class, more established area and a lower-income area with a higher concentration of recently-arrived immigrants), the consistently low attendance group may have been comprised primarily of recently-arrived immigrants whose ability to dedicate time to a family-based intervention may be more limited.

Like SES variables, neither weight status nor healthy lifestyle behaviors at baseline for either parents or adolescents distinguished between the attendance groups, despite indicating a high need for the intervention. Specifically, all adolescents and the majority of parents (86%) were in an unhealthy weight range. Adolescents self-reported engaging in 60 min/day of physical activity only about half the week and consuming less than 2 cups/day of fruits and vegetables. Similarly, parents self-reported consuming high amounts of added sugars (14.7 teaspoons/day). These findings suggested that when recruiting for an intervention such as Familias unidas for Health and Wellness, focusing on intervention health benefits and participants’ potential need may not be as important as focusing on culture. Alternatively, many of the non-significant findings in the current study, particularly those related to participants’ weight status and healthy lifestyle behaviors, may be explained by the largely homogenous sample that resulted from study recruitment and selection procedures (e.g., enrolling adolescents in the overweight or obese weight range only).

Acculturation variables, specifically parents’ Americanism and adolescents’ Hispanicism, were the only variables to distinguish among the three attendance patterns, highlighting the importance of taking acculturation into account as it relates to Hispanics’ participation in preventive interventions, e.g., References [26,28,30,44]. Acculturation is comprised of two dimensions (receiving culture acquisition, heritage culture retention), each of which occur in three domains (practices, values, identifications) [68]. Findings from the current study indicated that parents with lower receiving culture acquisition (i.e., lower Americanism) and adolescents with lower heritage culture retention (i.e., lower Hispanicism) were the most likely to belong to the consistently high attendance group. Importantly, 90% of parents and approximately 40% of adolescents enrolled in the intervention were immigrants from Latin American countries including Cuba, Nicaragua, Honduras, and Venezuela. Previous research suggests that culturally relevant interventions may present immigrant parents with opportunities to better understand numerous aspects of their host culture (e.g., parenting in the U.S., education and the school system) and simultaneously forge new social connections with other culturally similar adults [69]. It is therefore possible that parents viewed the intervention as an opportunity to connect with other similar adults, better understand American practices and values, and reconnect their children with their heritage culture. Similarly, it is possible that adolescents viewed the intervention as an opportunity to connect with other similar adolescents and for parents to understand their perspective.

This study has several limitations. Although the sample of Hispanics is diverse and represents immigrants from numerous Latin American countries, all of them were recruited from a single geographical location (Miami, FL, USA), limiting the generalizability of findings. Measures of parent and adolescent healthy lifestyle behaviors, including physical activity, fruit and vegetable intake, and added sugar intake, were self-reported and thus subject to bias. Beyond being self-reported, our measure of dietary intake may have failed to accurately capture the healthfulness of many foods (e.g., cereals with varying grams of sugar). In addition, alpha reliability for the positive parenting measure in this sample was low. Finally, while attendance measured an important aspect of intervention participation, it did not capture how actively participants engaged with session content. A growing body of literature is examining predictors of intervention “responsiveness”, or “dynamic engagement”, which includes constructs such as attendance, satisfaction, completion of outside practice assignments, and active participation in sessions [40,70].

This study also has various notable strengths. It adds to a growing body of literature on attendance and retention within pediatric obesity interventions and highlights how a repeated measures latent class analysis can be used to conceptualize attendance over the course of an intervention. It also uses a large sample of Hispanic families participating in an ongoing efficacy trial to understand attendance in a population for whom previous studies have reported problems with intervention attendance, retention, and drop out. Finally, the study assessed relevant predictors of attendance patterns in both parents and children rather than just one or the other.

## 5. Conclusions

Examining attendance patterns and predictors thereof provides important information regarding when and for whom declines in attendance are likely to occur in pediatric obesity interventions. Although we explored associations between eco-developmental risk and protective factors ranging from the individual through the broader cultural level, cultural variables seemed to be the most relevant for distinguishing among subgroups of participant attendance patterns. For Hispanics in particular, acculturation (and specifically, levels of both parent and adolescent Americanism and Hispanicism) should be considered in determining session attendance and in formulating strategies for increasing it. The pediatric obesity prevention literature would benefit from future investigations that explore a range of variables that predict participant attendance patterns. Although not the focus of the present study, it is also important that future research in this area examine attendance patterns as predictors and/or mediators of intervention effects on targeted outcomes.

## Figures and Tables

**Table 1:** Participant Demographic Characteristics at Baseline.

Variable	Intervention Condition (*n* = 140)
Adolescents	
Female, *n* (%)	69 (49.3%)
Age, M ± SD	13.04 ± 0.87
Country of Origin/Birth, *n* (%)	
US	85 (60.7%)
Cuba	29 (20.7%)
Honduras	3 (2.1%)
Venezuela	5 (3.6%)
Others	18 (12.9%)
BMI (kg/m^2^), M ± SD	28.29 ± 4.44
BMI percentile, M ± SD	95.28 ± 3.67
Weight Status, *n* (%)	
Overweight (BMI percentile 85th–95th)	50 (35.7%)
Obese (BMI percentile ≥ 95th)	90 (64.3%)
Parents	
Female, *n* (%)	122 (87.1%)
Age, M ± SD	42.09 ± 6.30
Country of Origin/Birth, *n* (%)	
US	14 (10.0%)
Cuba	50 (35.7%)
Nicaragua	17 (12.1%)
Honduras	17 (12.1%)
Others	42 (30.0%)
BMI (kg/m^2^), M ± SD	30.99 ± 6.14
Weight Status, *n* (%)	
Normal weight (BMI 18.5–24.9)	18 (12.9%)
Overweight (BMI 25–29.9)	48 (34.3%)
Obese (BMI ≥ 30)	73 (52.1%)
Unknown	1 (0.7%)
Marital Status, *n* (%)	
Partnered	112 (80.0%)
Unpartnered	28 (20.0%)
Full-time employment, *n* (%)	72 (51.4%)
Annual Income, *n* (%)	
<$30,000	84 (60.0%)
$30,000–$49,999	29 (20.7%)
≥$50,000	21 (15.0%)
Not reported	6 (4.3%)

**Table 2:** Weekly Session Topics and Activities.

Session	Session Topic	Activities
Family Session 1	Engagement and Orientation	Discussion on helping parents protect adolescents from health risks (e.g., diabetes, heart disease) and risky behaviors
Group Session 1	Parent Involvement in Adolescent Worlds	Parent-only discussion on protecting youth from health risks and risky behaviors Adolescent-only group SPARK games Parent + adolescent cooking class
Group Session 2	Communication	Parent-only discussion on effective communication Adolescent-only group SPARK games Parent + adolescent park overview and obstacle course
Family Session 2	Family Communication	Guided discussions and role plays about adolescent lifestyle behaviors and risky behaviors
Group Session 3	Behavior Management	Parent-only discussion on effectively managing adolescent behavior Adolescent-only group SPARK games Parent + adolescent yoga class
Group Session 4	Monitoring of Peers	Parent-only discussion on effectively monitoring adolescents’ peers Adolescent-only group SPARK games Parent + adolescent dance activity
Group Session 5	Substance Use and Other Unhealthy Behaviors	Parent-only discussion on substance use and other unhealthy behaviors (e.g., sugary beverage intake, screen time) Adolescent-only group SPARK games Parent + adolescent cooking class
Family Session 3	Monitoring of Peers and Unhealthy Behaviors	Guided discussions and role plays about substance use and other unhealthy behaviors
Group Session 6	Parental Investment in School World	Parent-only discussion on adolescent school bonding, academic achievement, school wellness opportunities, and lunch opportunities Adolescent-only group SPARK games Parent + adolescent cooking class
Group Session 7	Adolescent Risky Sexual Behavior and HIV	Parent-only discussion on adolescent risky sex Adolescent-only group SPARK games Parent + adolescent Zumba class
Family Session 4	Adolescent Risky Sexual Behavior and HIV	Guided discussions and role plays about adolescent risky sex
Group Session 8	Prevention Every Day	Parent-only review of sessions and skills learned Adolescent-only group SPARK games Parent + adolescent healthy group party

**Table 3:** Model Fit Indices of Latent Class Analysis and Sample Sizes for 1- to 5-class Solutions.

Number of Classes	AIC	BIC	SABIC	Adj. LMR-LRT (*p*-Value)	Entropy	Class Size				
						1	2	3	4	5
1	1960.95	1996.25	1958.28			140				
2	1356.58	1430.12	1351.03	620.704 (0.00)	0.98	103	37			
3	1335.11	1431.58	1311.35	61.82 (0.01)	0.97	101	21	18		
4	1319.79	1455.72	1326.00	19.34 (0.09)	0.97	100	17	12	11	
5	1315.30	1474.15	1303.30	16.97 (0.17)	0.89	52	48	17	12	11

**Table 4:** Profiles of Three Classes of Attendance Patterns.

Variables	Total Sample *n* = 140	Consistently Low ^a^ *n* = 18	Moderate and Decreasing ^b^ *n* = 21	Consistently High ^c^ *n* = 101	F- or *χ*^2^ Value, df = 2 (Post-Hoc Test)
Adolescents					
Female (vs male)	69 (49.3%)	9 (50.0%)	12 (57.1%)	48 (47.5%)	0.65
BMI %ile	95.27 (3.67)	94.97 (4.01)	94.93 (4.31)	95.40 (3.49)	0.21
Physical activity (days/week ^d^)	3.71 (2.37)	4.17 (1.68)	3.86 (2.53)	3.58 (2.44)	0.51
Fruit and vegetable intake (cups/day)	1.71 (0.61)	1.65 (0.53)	1.60 (0.63)	1.75 (0.63)	0.61
Sugar intake (tsp/day)	3.89 (1.66)	4.46 (1.69)	3.64 (1.33)	3.83 (1.70)	1.35
Positive parenting	21.03 (5.22)	23.61 (4.36)	21.57 (5.90)	20.44 (5.10)	3.02
Parent–adolescent communication	75.21 (12.45)	76.55 (12.54)	76.70 (14.49)	74.64 (12.06)	0.35
Hispanicism	37.94 (8.74)	41.50 (8.24)	40.19 (8.24)	36.84 (8.56)	3.07 * (c < a = b) *p* = 0.036
Americanism	48.03 (6.88)	49.94 (5.80)	48.71 (7.06)	47 .52 (7.00)	1.06
Parents					
Education	12.47 (3.18)	12.38 (3.43)	12.47 (2.50)	12.49 (3.29)	0.01
Annual Household Income (<$25,000)	73 (54.5%)	10 (58.8%)	9 (42.9%)	54 (56.3%)	1.39
Partnered (vs unpartnered)	80 (57.1%)	9 (50.0%)	9 (42.9%)	62 (61.4%)	2.87
Body mass index (BMI)	30.99 (6.14)	30.23 (5.04)	30.66 (6.34)	31.20 (6.31)	0.22
Physical activity (days/week ^d^)	3.48 (2.51)	3.59 (2.37)	3.90 (2.27)	3.37 (2.59)	0.38
Fruit and vegetable intake (cups/day)	2.67 (1.34)	2.74 (1.27)	3.19 (1.52)	2.56 (1.31)	1.89
Sugar intake (tsp/day)	14.66 (11.47)	18.79 (20.01)	15.59 (12.18)	13.74 (9.01)	1.57
Positive parenting	24.05 (4.29)	24.72 (4.68)	25.15 (3.78)	23.72 (4.31)	1.17
Parent–adolescent communication	78.13 (10.98)	75.83 (9.83)	83.10 (9.19)	77.55 (11.31)	2.64
Hispanicism	48.52 (5.98)	51.67 (4.27)	46.71 (7.94)	48.42 (5.66)	2.80
Americanism	36.92 (11.99)	37.08 (12.70)	43.42 (10.73)	35.54 (11.78)	3.91 * (c < b = a) *p* = 0.006

Values presented as M (SD) or *n* (%); ^a^ = Consistently low attendance; ^b^ = Moderate and decreasing attendance; ^c^ = Consistently high attendance; ^d^ = at least 60 min of physical activity/day; * *p* < 0.05.
